# Genotype-associated structural and nanomechanical alterations of aortic fibrillin-1 microfibrils in Marfan syndrome

**DOI:** 10.1016/j.mbplus.2026.100205

**Published:** 2026-08-31

**Authors:** Cristina M. Șulea, Dominik Sziklai, Bálint Kiss, Kálmán Benke, Miklós Pólos, Bence Ágg, Roland Stengl, Máté Csonka, Zoltán Szabolcs, Miklós S.Z. Kellermayer

**Affiliations:** a Department of Biophysics and Radiation Biology, Semmelweis University, 1094 Budapest, Hungary; b Heart and Vascular Center, Semmelweis University, 1122 Budapest, Hungary; c Hungarian Marfan Foundation, 1122 Budapest, Hungary; d HUN-REN-SE Biophysical Virology Group, 1094 Budapest, Hungary; e Department of Pharmacology and Pharmacotherapy, Semmelweis University, 1089 Budapest, Hungary

**Keywords:** Marfan syndrome, Fibrillin-1 microfibrils, Extracellular matrix, Inherited connective tissue disorders, Cardiovascular disease, Atomic force microscopy, Force spectroscopy

## Abstract

Marfan syndrome (MFS) is an autosomal dominant connective tissue disorder caused by mutations in the gene encoding fibrillin-1 (*FBN1*), the main component of extracellular microfibrils. In the aortic wall, these microfibrils maintain structural integrity and sustain hemodynamic load. Pathogenic *FBN1* variants are thought to structurally and functionally impair fibrillin-1 microfibrils, leading to progressive aortic aneurysm and dissection, the major causes of morbidity and mortality in MFS. However, the molecular mechanisms whereby these genetic variants translate into structural and mechanical defects are far from being understood.

Here we explored the morphology and the nanomechanical characteristics of individual aortic fibrillin-1 microfibrils from MFS patients and non-MFS controls by atomic force microscopy. The topographical assessment revealed a preserved overall pattern and periodicity of the microfibrils, but with morphological irregularities in MFS microfibril beads and interbead segments, consistent with presumed structural fragility. Force spectroscopy revealed a reduction of transverse elastic modulus in patients harboring haploinsufficient *FBN1* variants. Nanoindentation analysis was indicative of localized deformation, occurring at markedly lower forces in MFS microfibril beads, suggesting diminished load-bearing capacity.

These data provide direct nanoscale evidence of structural and mechanical consequences of *FBN1* mutations on human aortic tissue. Altered fibrillin-1 microfibril morphology and reduced stiffness in MFS support a pathogenetic mechanism in which compromised microfibrillar integrity weakens the aortic wall, predisposing it to progressive dilation.

## Highlights

• Human MFS and control aortic fibrillin-1 microfibrils were characterized with AFM.
• MFS microfibrils have preserved periodicity but display morphological alterations.
• Haploinsufficient *FBN1* variants lead to reduced microfibrillar transverse stiffness.
• MFS microfibrils exhibit bead-level mechanical failure at lower threshold forces.

## Introduction

Marfan syndrome (MFS) is the most prevalent and best-characterized fibrillinopathy, affecting around 1 in 5000 individuals worldwide [1]. It results from pathogenic variants in the fibrillin-1 gene (*FBN1*), of which roughly 25% arise de novo*,* while most follow autosomal dominant inheritance [2]. More than 3000 *FBN1* mutations have been reported, leading to molecular dysfunction through either dominant negative (DN) or haploinsufficiency (HI) mechanisms [3]. DN effects typically result from missense variants that introduce single amino acid substitutions, producing structurally abnormal fibrillin-1 monomers that impair microfibril assembly and function [4], [5]. DN mutations are frequently subclassified by cysteine involvement, as these amino acids are critical for molecular stability and supramolecular organization through the formation of structure-consolidating disulfide bridges [6]. In contrast, HI variants reduce functional fibrillin-1 levels, thereby impairing microfibril synthesis [7], [8].

Fibrillin-1 is a large (320 kDa), cysteine-rich glycoprotein central to extracellular matrix (ECM) architecture [9]. Its modular organization is dominated by calcium-binding epidermal growth factor (cbEGF)-like repeats and transforming growth factor-beta (TGF-β)-binding protein-like (TB) domains. At the supramolecular level, fibrillin-1 monomers assemble into beaded microfibrils with a characteristic periodicity of ∼56 nm and a repeat mass of around 2.55 MDa [10]. Microfibril diameter ranges from 10–12 nm reported in early transmission electron microscopy studies to 30–40 nm in atomic force microscopy (AFM) analyses, reflecting tissue-specific and pathological variability due to diabetes mellitus, photoaging, or smoking, among others [11], [12], [13], [14]. Microfibrils consist of alternating beads and interbead segments, and contain eight parallel fibrillin-1 molecules in cross-section [15], [16]. The precise monomer arrangement remains debated, however, with proposed configurations involving N- and C-terminal interactions within the bead region [17].

Mechanically, microfibrils provide elasticity and resilience to connective tissues. They can reversibly elongate from 56 nm up to 90 nm through TB-cbEGF interdomain sliding, whereas greater extension induces irreversible conformational changes at the bead level [18], [19]. Reported Young's modulus values vary widely (0.2–96 MPa), reflecting both experimental differences and biological variability [20]. Given their central role in maintaining the structural integrity of connective tissues, altered microfibrillar mechanics may critically influence tissue resilience and modulate the phenotypic consequences of *FBN1* defects.

Beyond their structural role, fibrillin-1 microfibrils regulate TGF-β signaling [21], [22]. By sequestering latent TGF-β complexes within the ECM, fibrillin-1 restricts cytokine activation until triggered by proteolysis, integrin binding, or mechanical strain [23]. Pathogenic *FBN1* variants can disrupt this regulatory function and alter TGF-β signaling, which has been implicated in MFS pathogenesis and aortic disease progression [21]. Elevated TGF-β activity has been associated with fibrosis and maladaptive remodeling in murine models and human studies [24], [25]. This biochemical dysregulation likely acts synergistically with mechanically weakened microfibrils and altered ECM organization, amplifying tissue vulnerability in MFS.

Although MFS displays variable clinical severity, primarily affecting the cardiovascular, respiratory, skeletal, and ocular systems, prognosis relies most heavily on aortic involvement [26], [27], [28]. Aortic root dilation, particularly at the sinuses of Valsalva, arises from medial degeneration involving ECM disorganization and altered biological responses, with TGF-β dysregulation acting as an exacerbating factor [29], [30]. Hemodynamic stress combined with structural wall fragility further promote aortic dilation, which begins in childhood and affects over 90% of patients during their lifetime [31]. Aortic growth rates vary with *FBN1* variant class and modifying factors [32], [33]. Aortic dissection occurs at younger age in MFS and accounts for approximately 5% of all cases, frequently leading to fatal rupture and/or valvular or myocardial complications if left untreated [34], [35].

Despite extensive biochemical and clinical characterization, the nanoscale architecture and mechanical behavior of fibrillin-1 microfibrils of human origin remain largely unexplored. We recently identified morphological alterations in MFS microfibrils [36]; in the present study, we investigated the nanomechanical properties of MFS fibrillin-1 microfibrils and explored their association with structural parameters and *FBN1* variant class.

## Results

### Study population overview

**Fig. 1:**
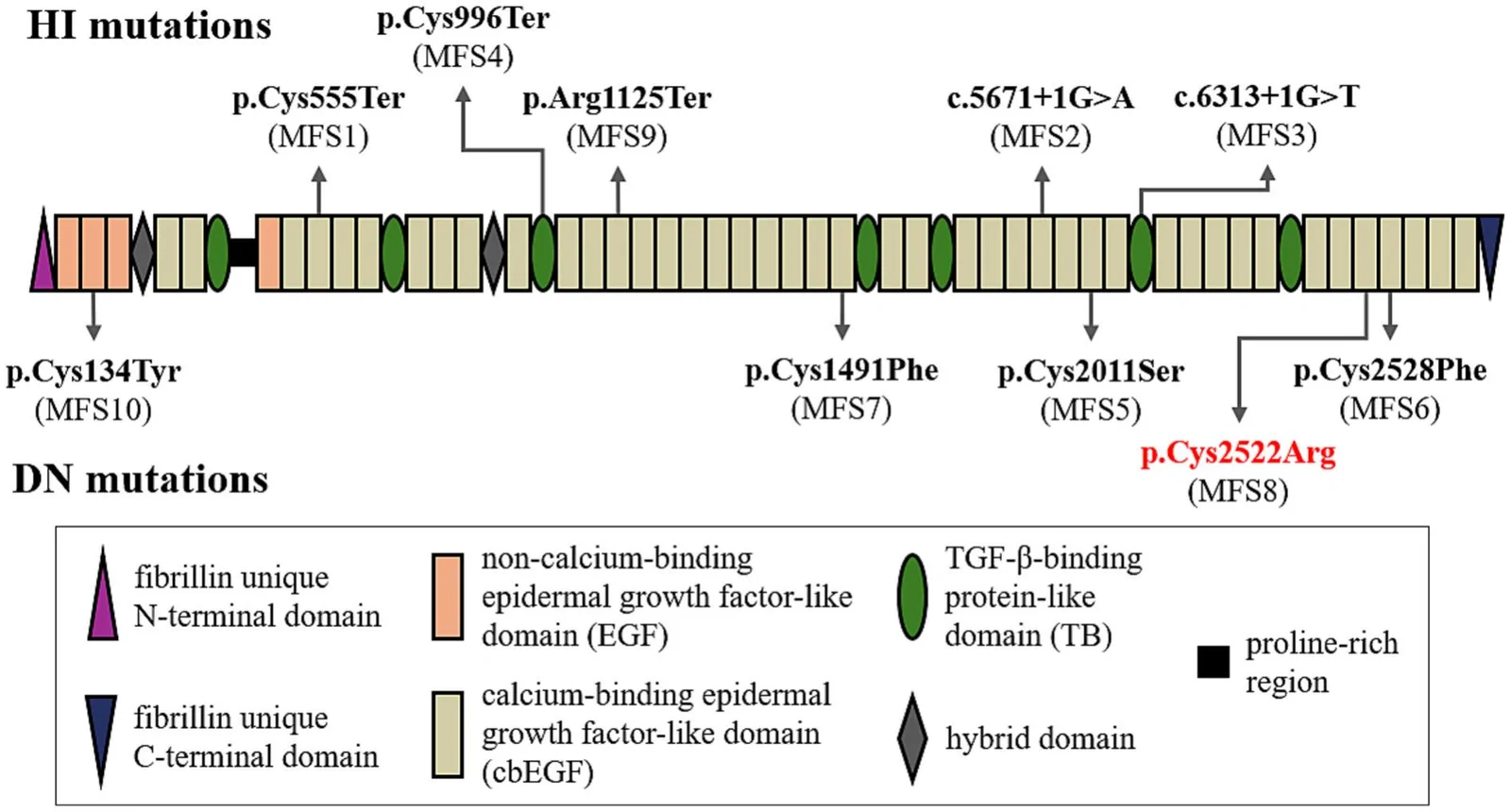
*FBN1* variants identified in the MFS cohort, mapped onto the domain organization of the fibrillin-1 protein and categorized (above and below the domain layout) according to predicted pathogenic mechanism. The novel variant is highlighted in red. (For interpretation of the references to colour in this figure legend, the reader is referred to the web version of this article.)

A heterogeneous spectrum of (likely) pathogenic *FBN1* variants was identified in the MFS cohort, comprising five missense, three nonsense, and two splice-site mutations (Table 1). Four variants were presumed de novo, including one previously unreported missense change. This variant (c.7564_7566delinsAGA) was identified in patient MFS8, a 50-year-old male fulfilling the diagnostic criteria for MFS based on aortic involvement and molecular confirmation, but without sufficient systemic features, *ectopia lentis*, or a positive family history. The localization of each variant within the fibrillin-1 domain structure is illustrated in Fig. 1.

**Table 1:** List of *FBN1* variants identified in the MFS cohort. Abbreviations: (cb) EGF, (calcium-binding) epidermal growth factor-like domain; DN, dominant negative; HI, haploinsufficiency; MFS, Marfan syndrome; TB, transforming growth factor-beta-binding protein-like domain.

Patient ID	Nucleotide change	Exon	Amino acid substitution	Protein domain	Mutation effect	Mutation type	Variant classification
**MFS1**	c.1665C > A	13	p.Cys555Ter	cbEGF4	nonsense	HI	pathogenic
**MFS2**	c.5671 + 1G > A	intron 46	–	cbEGF28	splice site	HI	pathogenic
**MFS3**	c.6313 + 1G > T	intron 51	–	TB6	splice site	HI	pathogenic
**MFS4**	c.2988 T > A	25	p.Cys996Ter	TB5	nonsense	HI	likely pathogenic
**MFS5**	c.6032G > C	49	p.Cys2011Ser	cbEGF30	missense	DN	pathogenic
**MFS6**	c.7583G > T	62	p.Cys2528Phe	cbEGF40	missense	DN	pathogenic
**MFS7**	c.4472G > T	37	p.Cys1491Phe	cbEGF22	missense	DN	likely pathogenic
**MFS8**	c.7564_7566delinsAGA	61	p.Cys2522Arg	cbEGF39	missense	DN	likely pathogenic
**MFS9**	c.3373C > T	28	p.Arg1125Ter	cbEGF13	nonsense	HI	pathogenic
**MFS10**	c.401G > A	5	p.Cys134Tyr	EGF2	missense	DN	pathogenic

MFS patients were stratified according to the predicted molecular consequence into HI (*n* = 5) and DN (*n* = 5) groups, as previously described [6]. Comparative analyses were performed between mutation classes and against controls.

The demographic and clinical characteristics of the study population are summarized in Table 2. Overall, individuals with MFS were significantly younger than controls (34.1 ± 12.7 years vs. 56.5 ± 7.0 years, *p* < 0.001) and had a lower body mass index (BMI) (23.3 ± 5.9 kg/m^2^ vs. 27.5 ± 2.7 kg/m^2^, *p* = 0.026). The control group exhibited a higher prevalence of hyperlipidaemia (*p* = 0.006) and diabetes mellitus (*p* = 0.033), while no significant differences were found for hypertension (*p* = 0.087), smoking (*p* = 0.350), or obesity, defined as BMI > 30.0 (*p* > 0.999). In the MFS group, the mean diameters of the aortic annulus, sinuses of Valsalva, and ascending aorta were 24.9 ± 2.9 mm, 49.6 ± 8.8 mm, and 42.7 ± 8.0 mm, respectively. Comprehensive preoperative echocardiographic data were unavailable for most control patients due to absent or incomplete imaging records. Consequently, only the aortic annulus and ascending aorta diameters could be evaluated in this group, with mean values of 23.8 ± 5.7 mm and 31.5 ± 4.6 mm, respectively. Ascending aortic diameter was significantly larger in patients with MFS than in controls (*p* = 0.005). Individual demographic and clinical parameters of all study participants are provided in Supplementary Tables S1 and S2.

**Table 2:** Baseline demographic and clinical characteristics of the study cohorts. Abbreviations: BMI, body mass index; DN, dominant negative; HI, haploinsufficiency; MFS, Marfan syndrome; NA, not available/not applicable.

**Studied characteristics**	**HI** *n* = 5	**DN** n = 5	**Control** *n* = 10
**Age (years)**	37.6 ± 13.6	30.6 ± 12.2	56.5 ± 7.0
**Sex (male)**	2 (40%)	5 (100%)	7 (70%)
**BMI (kg/m**^**2**^**)**	23.5 ± 4.5	23.2 ± 7.6	27.5 ± 2.7

**Risk factors for atherosclerosis (n, frequency)**			
**Hypertension**	2 (40%)	4 (80%)	10 (100%)
**Hyperlipidemia**	0	1 (20%)	8 (80%)
**Diabetes mellitus**	0	0	5 (50%)
**Smoking**	0	2 (40%)	5 (50%)
**Obesity**	0	1 (20%)	2 (20%)

**Preoperative echocardiographic findings (mm)**			
**Aortic root diameter**	23.2 ± 2.6	27.0 ± 1.8	23.8 ± 5.7
**Sinus Valsalva diameter**	47.0 ± 3.5	52.2 ± 12.0	NA
**Ascending aortic diameter**	41.2 ± 9.7	44.5 ± 5.9	31.5 ± 4.6

**MFS-specific diagnostic criteria according to the revised Ghent nosology [26] (n, frequency)**			
***FBN1* mutation**	5 (100%)	5 (100%)	NA
**Family history**	4 (80%)	2 (40%)	NA
**Aortic involvement**	5 (100%)	5 (100%)	NA
***Ectopia lentis***	2 (40%)	2 (40%)	NA
**Systemic involvement**	5 (100%)	3 (60%)	NA

### Morphological characterization of aortic fibrillin-1 microfibrils

**Fig. 2:**
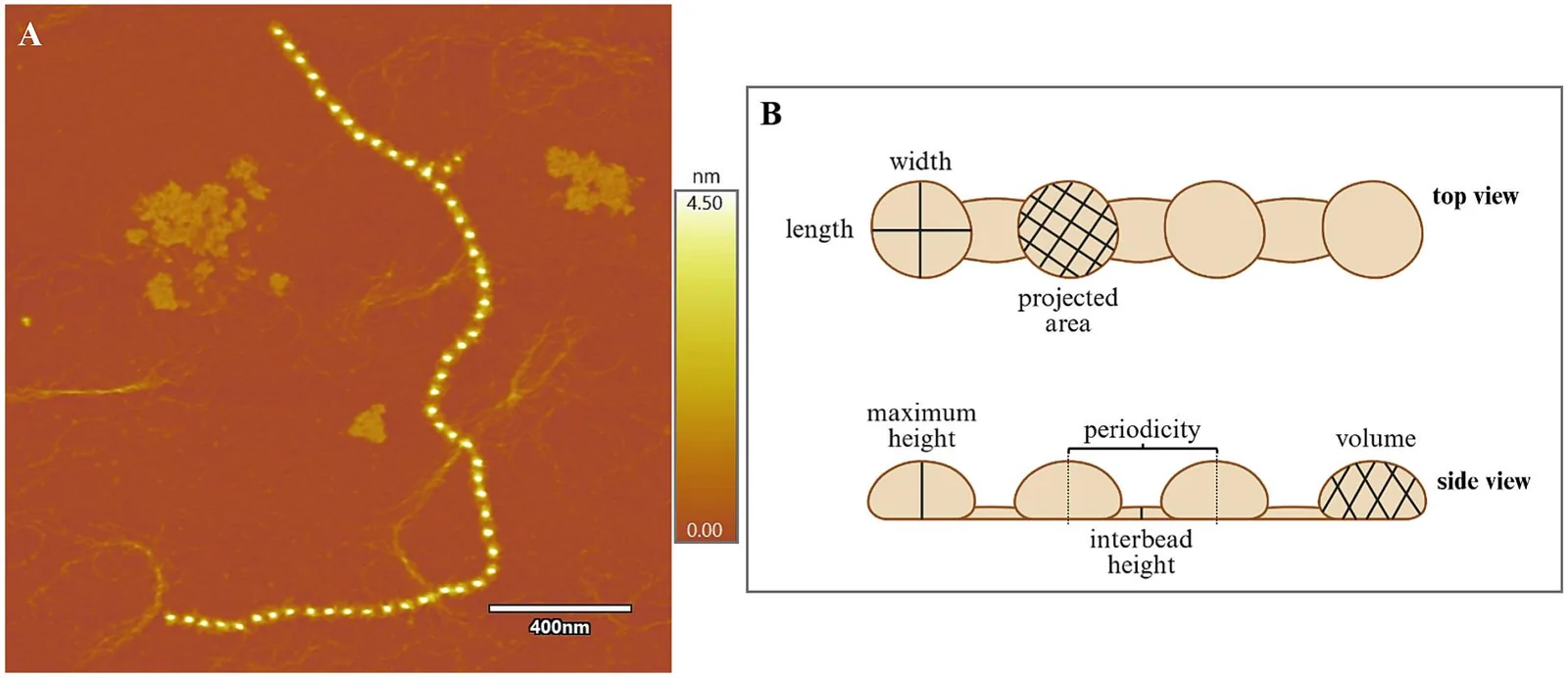
**A.** Representative height-contrast AFM image of an aortic fibrillin-1 microfibril isolated from human aortic tissue (sample MFS1). **B.** Schematic illustrating the geometric parameters used for quantitative morphological analysis of fibrillin-1 microfibrils.

As previously reported, AFM imaging revealed fibrillin-1 microfibrils exhibiting the characteristic “beads-on-a-string” appearance in all samples (Fig. 2, Supplementary Figs. S2 and S3) [36]. For quantitative analysis, only linear and isolated microfibrils were included, yielding 174 structures comprising a total of 4333 beads. Detailed morphometric data for each sample are provided in Supplementary Tables S3 and S4.

**Fig. 3:**
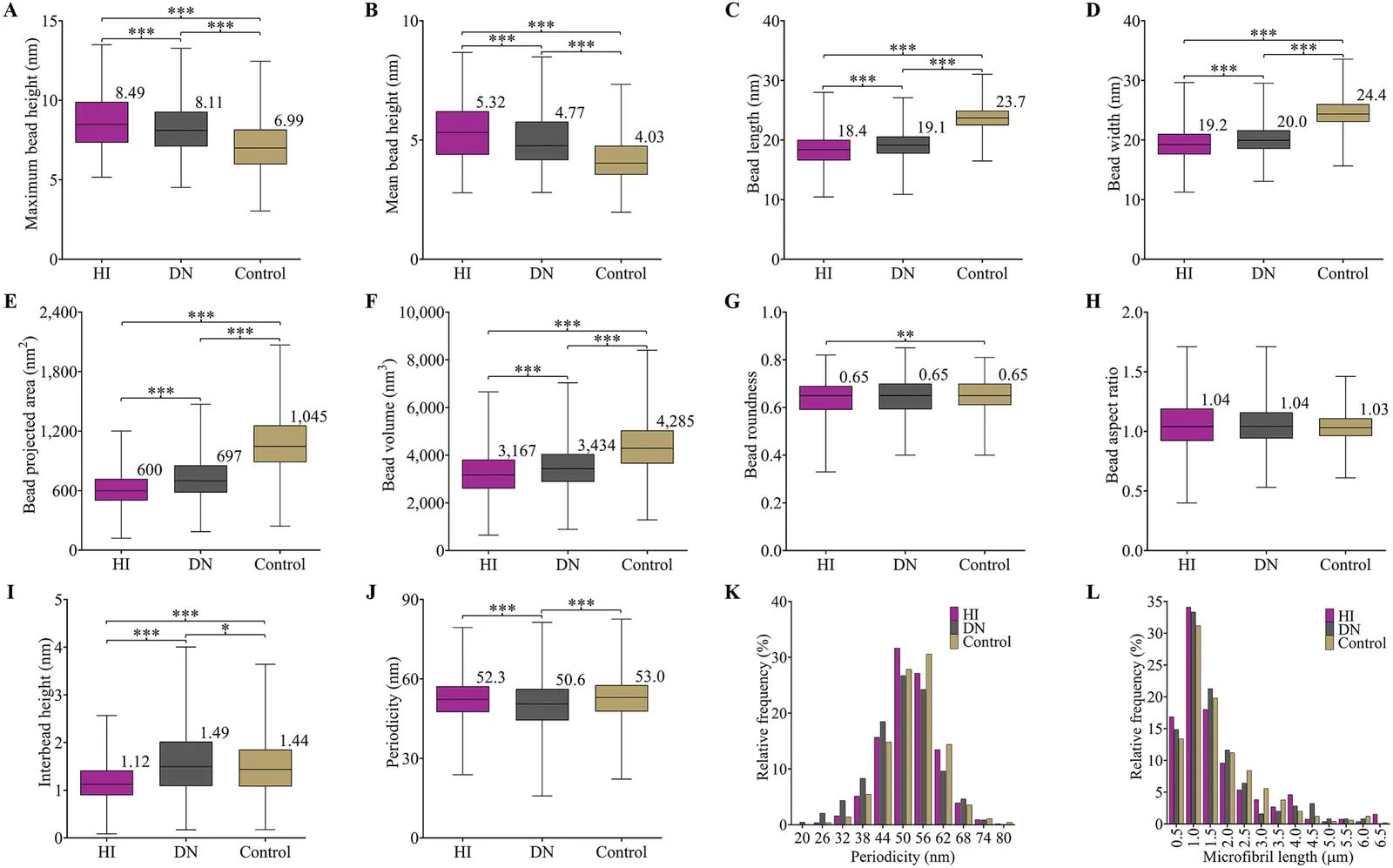
Quantitative morphological characterization of HI, DN and control fibrillin-1 microfibrils: maximum (**A**) and mean (**B**) bead height, axial length (**C**), width (**D**), projected area (**E**), volume (**F**), roundness (**G**), and aspect ratio (**H**), maximum interbead height (**I**), and microfibril periodicity (**J**). Box plots indicate the median (horizontal line), interquartile range (box), and range (whiskers); median values are shown adjacent to each box. Analyses were performed on 1084, 1110, and 2129 beads from the HI, DN, and control groups, respectively. Panels **K**–**L** present the distribution profiles of microfibril periodicity and length across the investigated groups. Microfibril length measurements were obtained from 261, 249, and 500 microfibrils in the HI, DN, and control groups, respectively. Statistical significance: *p < 0.05, **p < 0.01, ***p < 0.001.

Overall, microfibrils isolated from MFS aortic tissue displayed increased morphological heterogeneity and consistent deviations from controls. Compared with HI samples, DN beads demonstrated reduced height but greater lateral dimensions, resulting in larger projected area and volume (Fig. 3A–F). Nevertheless, both MFS groups showed reduced bead dimensions relative to controls, with alterations being more pronounced in the HI group.

Analysis of bead shape descriptors (roundness and aspect ratio) indicated a slightly elongated bead profile of human aortic fibrillin-1 microfibrils, with seemingly comparable values across groups (Fig. 3G, H). The beads displayed a laterally ellipsoidal geometry, with mean dimensions of 8.5 × 18.4 × 19.2 nm in HI, 8.1 × 19.1 × 20.0 nm in DN, and 7.0 × 23.7 × 24.4 nm in control tissue.

Interbead height was significantly reduced in HI microfibrils compared with both DN and control groups (1.12 nm vs. 1.49 nm and 1.44 nm, respectively; *p* < 0.001), whereas DN samples showed values closer to controls (*p* = 0.030) (Fig. 3I). Microfibril periodicity clustered within the physiological range of 50–56 nm across all groups, irrespective of pathological status (Fig. 3J,K). A consistent inverse correlation between interbead height and periodicity was observed in all cohorts (Supplementary Table S4). Microfibril length varied substantially, from short assemblies comprising a few bead repeats to filaments extending up to 15 μm, based on analysis of 1010 filaments (Fig. 3L).

### Nanomechanical investigation

**Fig. 4:**
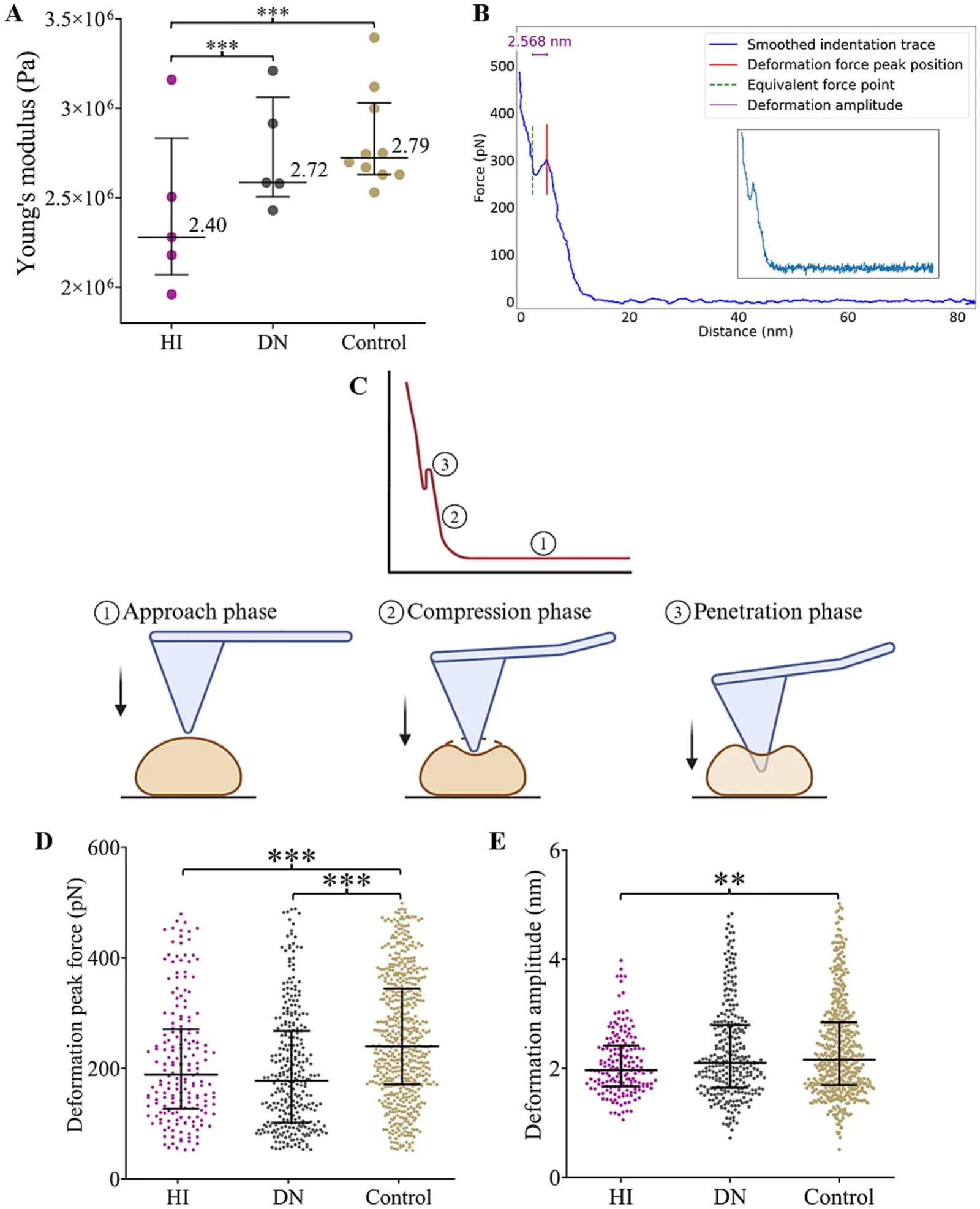
Nanomechanical investigation of human aortic fibrillin-1 microfibrils. **A.** Distribution of median Young's modulus values in HI, DN, and control groups. Bars represent medians (shown) and IQRs. Individual values are presented in Supplementary Table S5. **B.** Representative Python-processed force-distance curve illustrating the smoothed approach phase during AFM indentation. Deformation amplitude calculation is exemplified in purple. The inset shows the raw curve prior to data smoothing. **C.** Schematic representation of the proposed mechanical sequence during indentation: (1) approach of the AFM tip toward the bead; (2) progressive deformation with gradual increase in force; (3) abrupt force drop corresponding to tip entry or structural failure of the bead. Created in https://BioRender.com. **D.** Distribution of deformation peak forces in HI, DN, and control groups. **E.** Distribution of deformation amplitude values across HI, DN, and control microfibrils. Statistical significance: **p < 0.01, ***p < 0.001. (For interpretation of the references to colour in this figure legend, the reader is referred to the web version of this article.)

A total of 3930 beads derived from 281 fibrillin-1 microfibrils were analyzed on the basis of more than 7500 force-distance curves acquired by AFM force mapping. In control human aortic samples, Young's modulus values ranged from 2.53 to 3.40 MPa. HI microfibrils demonstrated significantly reduced median stiffness compared with both DN and control groups (*p* < 0.001), whereas no significant difference was detected between DN and control samples (*p* = 0.327) (Fig. 4A). Median Young's modulus values and distributions for each sample are provided as Supplementary Materials (Table S1 and Fig. S1).

The force-distance curves displayed abrupt drops in the recorded force signal of the indentation trace in a subset of measurements, interpreted as localized disruption events (mechanically-driven structural transitions within the manipulated beads) (Fig. 4B,C). Median deformation peak forces were 240 pN (171–344) in controls, 188 pN (127–271) in HI, and 178 pN (102–268) in DN microfibrils. Both MFS groups displayed significantly lower values than controls (*P* < 0.001), but were not different from each other (*p* = 0.369) (Fig. 4D). Deformation amplitudes measured 1.97 nm (1.67–2.42), 2.10 nm (1.65–2.79), and 2.16 nm (1.69–2.84) in HI, DN, and control microfibrils, respectively. HI values were significantly reduced compared with controls (*p* = 0.008) (Fig. 4E). Individual data are summarized in Supplementary Table S5.

## Discussion

We hypothesized that ultrastructural and mechanical alterations in fibrillin-1 microfibrils contribute to aortic root pathology in MFS. Accordingly, we performed AFM investigations to quantify bead and interbead morphology as markers of microfibrillar organization, molecular packing, and surface topography. In parallel, AFM force spectroscopy was applied to assess transverse stiffness, thereby providing insight into the impact of *FBN1* mutations on microfibril elasticity. Building upon our previous work [36], this study advances the understanding of fibrillin microfibril pathology by integrating comprehensive structural and nanomechanical analyses with genotype-specific stratification, thereby establishing a direct link between pathogenic *FBN1* variation and extracellular matrix architecture at the nanoscale.

The MFS cohort displayed marked allelic heterogeneity; seven of the included *FBN1* variants involved cysteine residues, primarily within cbEGF and TB domains, in which conserved cysteines form disulfide bonds critical for fibrillin-1 folding and structural stability [37], [38]. Cysteine substitutions are therefore expected to disrupt domain conformation, whereas nonsense and splice-site variants are predicted to result in HI via transcript degradation or aberrant splicing [39]. This broad spectrum of anticipated molecular effects, ranging from localized domain destabilization to reduced protein abundance, provides a mechanistic framework for the altered morphology and diminished mechanical resilience observed in MFS-derived fibrillin-1 microfibrils.

### Aortic fibrillin-1 microfibril morphology in MFS

Our findings demonstrated consistent morphological deviations in MFS microfibrils compared with controls, in line with our previous observations [36]. In HI samples, bead dimensions were consistently reduced, suggesting a disrupted microfibrillar organization. DN microfibrils exhibited intermediate dimensions, possibly reflecting mutation-specific structural effects. These differences were best captured by bead volume, a sensitive surrogate of overall three-dimensional organization, which followed the hierarchy HI < DN < control (3167 vs. 3434 vs. 4285 nm^3^). While bead heights were comparable across groups and consistent with prior AFM studies [11], [40], lateral dimensions (18–25 nm) were slightly lower than in earlier reports (30–40 nm) [11], likely reflecting cantilever geometry effects. Although measurements were performed on dehydrated microfibrils, the low intragroup variability and the consistent intergroup differences support the robustness of the comparative analyses.

Interbead height displayed considerable variability among individual MFS samples and was significantly reduced in HI microfibrils, while remaining relatively uniform in controls. This heterogeneity likely reflects mutation-induced disruption of fibrillin-1 regions contributing to the interbead segment. In contrast, periodicity remained tightly distributed across groups, indicating preserved axial spacing despite local ultrastructural abnormalities. The inverse correlation between interbead height and periodicity indicates that microfibril elongation involves bead separation accompanied by interbead thinning, consistent with prior models proposing reversible TB–EGF domain unfolding at moderate extensions (approximately 90–100 nm) and irreversible bead unraveling at higher strains [18], [41]. The length of the isolated microfibrils was highly variable in both MFS and control tissues, suggesting that fragmentation during the isolation process occurs independently of genotype.

**Fig. 5:**
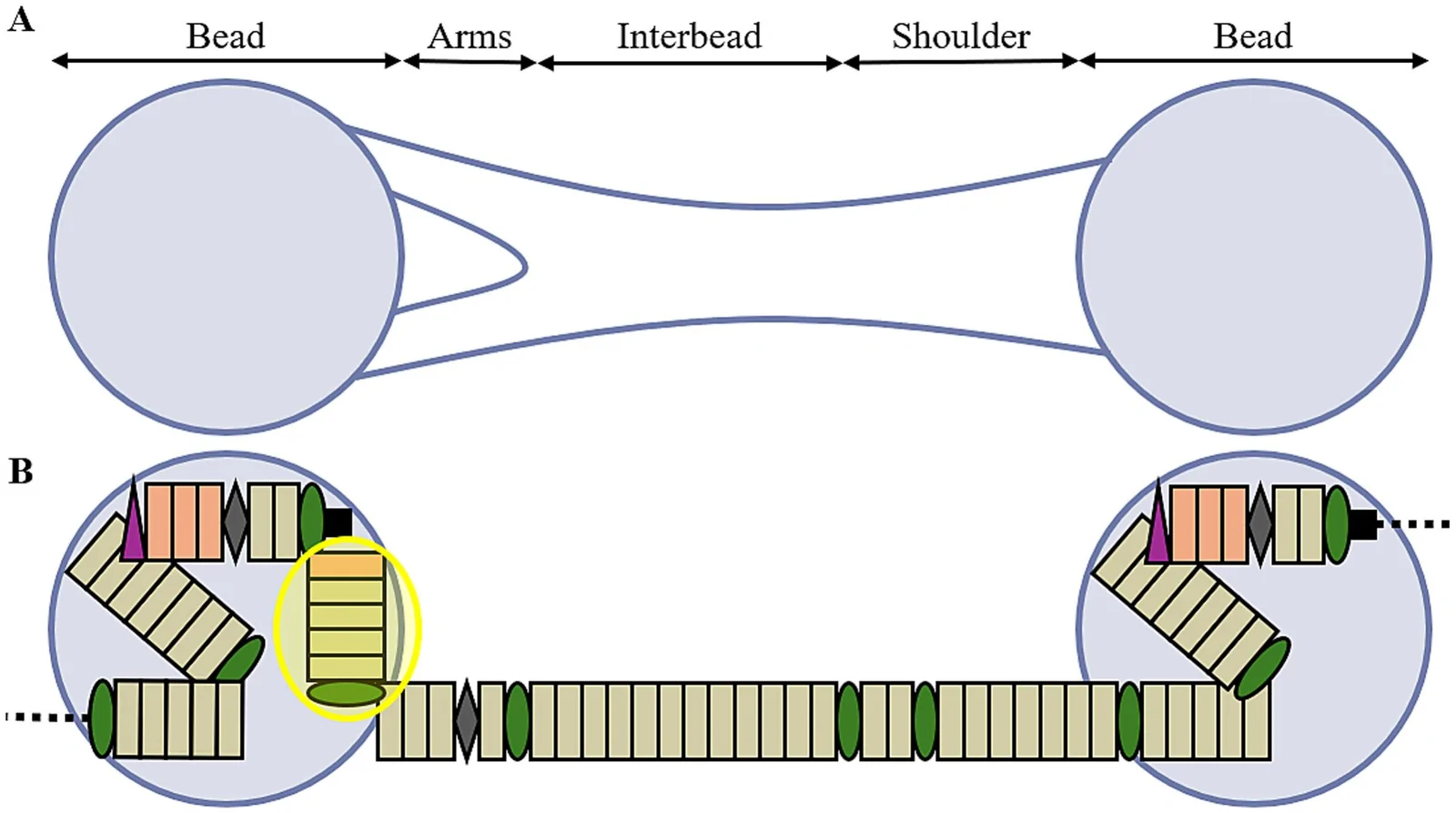
Schematics of molecular arrangement in fibrillin-1 microfibrils. **A.** Global structural features. Transmission electron microscopy has enabled subdivision of the regions connecting adjacent beads into arm, interbead, and shoulder segments. As these substructures cannot be reliably resolved by AFM, they were collectively defined as the interbead region in the present study. **B.** Proposed spatial organization of fibrillin-1 domains within a single microfibril repeat (adapted from [15]). Each repeat accommodates eight parallel fibrillin-1 molecules within the microfibril cross-section. Domains EGF4–TB2 (yellow ellipsoid) are buried within the densely packed bead core, whereas TB1 and the adjacent proline-rich region are positioned toward the bead periphery. Domains distal to TB2 up to TB3 extend to form the arm region, and TB4 localizes to the interbead-shoulder junction. The mass and volume of the bead further support extensive interweaving of both N- and C-terminal fibrillin-1 domains, suggesting that, following initial head-to-tail interactions, additional domains (likely including regions upstream of TB6) are incorporated into the bead structure. (For interpretation of the references to colour in this figure legend, the reader is referred to the web version of this article.)

Interpretation of these findings requires consideration of the underlying molecular consequences of individual *FBN1* mutations. However, the supramolecular organization of fibrillin-1 within the microfibril remains incompletely resolved, limiting definitive variant-specific conclusions. Structural models based on antibody mapping and cryogenic electron microscopy indicate that eight fibrillin-1 monomers are organized in a head-to-tail configuration, with substantial overlap of terminal regions within the bead and intervening domains contributing to the interbead region (Fig. 5) [15], [18], [42], [43]. Complementary studies combining AFM with proteomic analyses have demonstrated that fibrillin microfibrils exhibit tissue-specific differences in both morphology and protein composition despite maintaining a conserved ultrastructural organization. Microfibrils isolated from human skin and ocular zonules displayed distinct bead morphologies accompanied by differences in associated extracellular matrix proteins, including fibulins, microfibril-associated glycoproteins, laminins and perlecan [44]. Earlier proteomic characterization likewise established fibrillin-1 as the predominant structural component of mature microfibrils while identifying several associated proteins that likely contribute to their biological and mechanical properties [45]. These findings suggest that alterations in bead morphology may reflect changes in the molecular environment of the microfibril, in addition to differences in fibrillin-1 abundance.

All DN variants identified in this MFS cohort were missense substitutions affecting cysteine residues within cbEGF or EGF domains, thereby disrupting disulfide bonds essential for proper folding and molecular stability. Such alterations are expected to compromise domain conformation, perturb calcium-binding motifs, increase proteolytic susceptibility, and ultimately promote local misfolding and abnormal microfibril assembly [46], [47], [48]. In contrast, HI variants, predicted to induce nonsense-mediated mRNA decay, were associated with pronounced structural alterations despite the presumed predominance of wild-type fibrillin-1. This suggests that reduced protein availability alone may impair microfibril assembly [49]. Alternatively, partial incorporation of truncated monomers cannot be excluded, as residual mutant transcript levels ranging from 2% to 28% of wild-type have been reported [50], [51], [52]. Together with increased susceptibility to mechanical stress or proteolytic degradation [53], [54], these mechanisms likely underlie the thinner and structurally fragile microfibrils observed in HI samples.

Collectively, the morphological abnormalities observed in both HI and cysteine-altering DN microfibrils indicate that *FBN1* variants disrupt microfibril architecture irrespective of the underlying molecular mechanism, consistent with the comparably severe aortic phenotypes described for both mutation classes [55]. Although the control cohort comprised older individuals with a higher prevalence of cardiovascular risk factors, their microfibrillar parameters approximated earlier imaging data and exhibited lower intragroup variability, supporting their validity as reference samples. Therefore, the alterations detected in MFS microfibrils are likely attributable to mutation-driven effects rather than age- or disease-related influences, or technical artifacts. While AFM imaging cannot distinguish between reduced protein abundance, altered associated protein composition, or structural misfolding as primary causes, the more pronounced deviations in bead volume and interbead height observed in HI samples favor decreased fibrillin-1 availability as the predominant mechanism.

### Nanomechanics of fibrillin-1 microfibrils in the MFS aorta

The Young's modulus values obtained for human aortic fibrillin-1 microfibrils (1.96–3.21 MPa in MFS and 2.53–3.40 MPa in controls) were slightly higher than previously reported stiffness measurements for fibrillin-1-rich structures across various species and tissues, including sea cucumber dermis (0.2 MPa) [56], jellyfish mesogleal fibers (0.9 MPa) [20], porcine and octopus aorta (0.4 MPa) [57], [58], and bovine zonular fibers (0.19–1.88 MPa) [59]. An alternative methodology applied to bovine zonular fibrillin-1 microfibrils produced values two orders of magnitude greater (78–96 MPa) [19]. Nevertheless, the differences in species, tissue origin, and experimental setup limit direct comparisons. These discrepancies likely also reflect differences in hierarchical organization and loading modality, as tensile approaches assess axial stiffness, whereas AFM indentation measures local deformation resistance. Therefore, fibrillin-1 nanomechanical properties should be interpreted in the context of the specific experimental approach used.

In our experiments, MFS microfibrils displayed a broader distribution of Young's moduli and overall reduced stiffness relative to controls. HI microfibrils had significantly lower stiffness than both DN and control samples, whereas DN values did not differ significantly from controls. The narrower distribution in the control group suggests a stable mechanical profile characteristic of normal aortic fibrillin-1 microfibrils. When considered alongside the aforementioned structural data, the reduced stiffness in HI microfibrils likely reflects diminished protein incorporation, resulting in more compliant beads. This may arise from limited incorporation of wild-type monomers due to quantitative deficiency or partial incorporation of truncated mutant monomers contributing less structural mass [50], [60]. By contrast, DN microfibrils retained overall stiffness despite modest reductions in bead dimensions, suggesting local packing defects attributable to missense-induced misfolding, particularly involving disulfide bonds or calcium-binding motifs [61]. Such alterations may also perturb interactions with fibrillin-associated proteins, thereby modifying bead morphology without markedly affecting bulk stiffness [15].

Approximately 7–40% of the recorded force-distance curves exhibited sawtooth-like deflections in the indentation phase, interpreted as force-induced localized structural transition events within individual beads. Both HI and DN groups demonstrated similarly reduced deformation forces relative to controls, potentially suggesting a shared vulnerability to mechanical failure. This may reflect impaired domain folding, altered interdomain sliding, or reduced stability of fibrillin-1 supramolecular packing, as previously described for some MFS-causing variants [62], [63]. Notably, the deformation amplitude was approximately 2 nm. This dimension corresponds closely to the reported width of cbEGF domains and of the fibrillin-1 molecule itself [64], [65], raising the possibility that during indentation the AFM cantilever tip displaces the intertwined fibrillin-1 monomers within the bead, inducing partial unfolding, local rearrangement, or slippage of individual fibrillin-1 segments within the densely packed structure. Such events may occur at lower forces in MFS due to compromised supramolecular organization.

The magnitude of these force transitions is comparable with forces reported for mechanical transitions in modular extracellular matrix proteins, where unfolding of fibronectin type III domains occurs at approximately 80–200 pN, and mechanically induced transitions in fibrillin-1 cbEGF domains have been predicted in the range of approximately 120–300 pN depending on calcium stabilization and structural context [66], [67]. Although the indentation forces measured here cannot be directly equated with single-domain unfolding forces, the reduced peak forces observed in MFS samples suggest decreased local mechanical stability of fibrillin-1 assemblies. These events likely reflect collective nanoscale rearrangements, including partial unfolding, interdomain sliding, or disruption of intermolecular packing within fibrillin-1 beads rather than rupture of individual molecular bonds. Such nanoscale events are difficult to resolve morphologically as fibrillin-1 molecules within beads form highly convoluted assemblies that cannot be easily distinguished by conventional AFM imaging.

In summary, these findings suggest that focal mechanical fragility in MFS microfibrils is driven primarily by failure-prone architectural features rather than by reduced elastic compliance alone. The apparent discrepancy observed in DN microfibrils – preserved global stiffness but reduced deformation force – further implies that maintained bulk mechanical properties do not preclude localized structural weakness resulting from aberrant molecular packing.

### Practical interpretation of results

Within the aortic media, fibrillin-1 microfibrils envelop elastin cores, reinforcing elastic fibers and potentially facilitating force transmission [57]. Elastin forms concentric *lamellae* with smooth muscle cells and collagen [68], enabling dynamic adaptation to pulsatile blood pressure, with fibrillin-1 microfibrils acting as load-bearing elements that support elastic recoil [69].

Our findings indicate that MFS microfibrils display reduced bead and interbead dimensions, decreased transverse stiffness, and lower resistance to mechanical stress. These alterations may increase susceptibility to microdamage accumulation and adaptive deformation under repetitive loading, thereby weakening elastic fiber support. Such impairment could facilitate lamellar fragmentation, elevate wall stress, and promote progressive aortic wall dilation, ultimately contributing to ECM degradation and smooth muscle cell loss, hallmarks of aortic pathology in MFS [70].

### Limitations

Several limitations of this study should be acknowledged. First, the relatively small cohort size reflects the inherent challenges of investigating rare genetic disorders such as MFS. Access to suitable human aortic tissue is restricted to clinically indicated surgical procedures, limiting opportunities for large-scale recruitment. Moreover, because tissue collection was only possible in patients undergoing aortic surgery, the MFS cohort represents individuals with more advanced aortic involvement requiring operative intervention. Consequently, the observed microfibril alterations may not fully reflect the spectrum of fibrillin-1 abnormalities present in patients with milder disease manifestations or earlier disease stages. In addition, all DN variants included in this study were cysteine-altering mutations, a genotype class that has been associated with a more severe cardiovascular phenotype. Therefore, the differences observed between HI and DN groups should be interpreted within the context of this specific mutation subset. Future studies incorporating larger cohorts with broader genotypic and phenotypic diversity will be required to determine the generalizability of these findings.

Another limitation is the lack of truly healthy control tissue. Cadaveric samples were avoided to preserve tissue quality and ensure consistent harvesting conditions, and controls were therefore derived from patients without known connective tissue or aortic disease. The older age of the control cohort reflects the challenge of obtaining ascending aortic tissue from younger individuals without aortic pathology. Although age-related effects cannot be excluded completely, no significant correlation was observed between age and median Young's modulus (Pearson *r* = 0.438, *p* = 0.053, *n* = 20), suggesting that age alone is unlikely to account for the observed nanomechanical differences. Differences in comorbidities or tissue condition may nevertheless have introduced subtle variability in the measured properties of fibrillin-1 microfibrils, but our relatively small sample size would make multivariable statistical analyses underpowered, so we instead state it as an honest limitation.

In summary, this study provides a detailed characterization of human aortic fibrillin-1 microfibrils, revealing marked morphological alterations in MFS that are consistent with disrupted molecular organization. Reduced transverse stiffness, particularly in HI variants, and the lower deformation force thresholds observed in MFS beads indicate an apparently diminished capacity to withstand mechanical stress. Collectively, these findings demonstrate that genetically distinct classes of *FBN1* variants converge on a common phenotype of impaired nanomechanical performance and increased microfibril fragility under load. By linking patient genotype to nanoscale fibrillin microfibril architecture and mechanics, this work provides new insight into the molecular basis of aortic disease in MFS.

## Materials and methods

### Experimental design

The study was conducted in accordance with the principles of the Declaration of Helsinki (1975, revised 2013) following national ethical committee approval (permission numbers: ETT TUKEB 7891/2012/EKU [119/PI/12], ETT TUKEB 12751–3/2017/EKU – reviewed under 06475–7/2021/EÜIG and NNGYK/39546–5/2025 –, and ETT TUKEB BM/17671–3/2024). Written informed consent was obtained from all participants prior to inclusion.

Aortic root tissue specimens were collected intraoperatively from ten patients with MFS (MFS1–MFS10) and ten non-MFS control subjects (C1–C10). All procedures were performed at the Heart and Vascular Center of Semmelweis University. Participants were required to be at least fifteen years of age. Inclusion criteria for the MFS group comprised a genetically confirmed (likely) pathogenic *FBN1* variant and an indication for aortic root surgery due to MFS-related aortic pathology (aortic root aneurysm or annuloaortic ectasia). Control subjects had no personal or family history of connective tissue or aortic disease and underwent orthotopic heart transplantation for ischemic cardiomyopathy.

The samples were excised from the region above the sinuses of Valsalva, immediately immersed in sterile 0.9% saline solution, and transported on ice to the Department of Biophysics and Radiation Biology of Semmelweis University. The specimens were stored at 4 °C and processed within 48 h of collection. Clinical data were retrieved from the Hungarian Marfan Register [71] and the Transplantation Biobank of the Heart and Vascular Center of Semmelweis University.

### Fibrillin-1 microfibril extraction

Fibrillin-1 microfibrils were isolated as previously described [36], relying on an adaptation of earlier methodologies [72], [73]. Briefly, homogenized aortic wall tissue was incubated for four hours at room temperature with gentle stirring in 1 mg/mL type 1 A bacterial collagenase (Sigma-Aldrich, C9891). The resulting extracts were sequentially prepared under low-salt (0.4 M NaCl) and high-salt (1 M NaCl) conditions in 0.05 M Tris-HCl buffer (pH 7.4) supplemented with 0.01% NaN_3_ and protease inhibitors. Both extracts were purified by size-exclusion chromatography on a Sepharose CL-2B column equilibrated with 0.05 M Tris-HCl buffer (pH 7.4) containing 0.4 M NaCl and 0.01% NaN_3_. Fractions with the highest protein concentration were pooled for subsequent AFM analysis. As high-salt extracts yielded a greater amount of fibrillin-1 microfibrils, all AFM measurements were conducted on fractions from this eluate.

### Morphological assessment

Topographic AFM imaging was performed according to an optimized version of our earlier protocol [36]. 20 μL of eluate suspension was deposited onto freshly cleaved mica, equilibrated for 15 min, rinsed with ultrapure water, and dried under a gentle nitrogen stream. Imaging was performed at room temperature using a Cypher ES AFM (Asylum Research, Oxford Instruments) in tapping mode with OMCL-AC160TS-R3 microcantilevers (Olympus Corporation; nominal force constant 26 N/m, resonance frequency in air 300 ± 100 kHz). Scans were acquired at a 700 mV setpoint and 512 × 512 pixel resolution.

Initial morphometric measurements were performed in Igor Pro 6 (WaveMetrics, Lake Oswego, OR) along two orthogonal axes and included bead length and width (quantified as full width at half maximum of the corresponding height profiles), interbead height, periodicity, and total microfibril length [36]. For advanced morphological characterization, Gwyddion [74] was used to segment individual beads via Otsu's thresholding, ensuring objective selection. From each segmented bead, maximum and mean height, projected area, volume, roundness (ratio of the largest inscribed to smallest circumscribed circle diameters), and aspect ratio (width-to-length ratio) were extracted to provide a detailed quantitative assessment of bead geometry.

### Nanomechanical investigation

AFM force spectroscopy was performed in liquid (chromatography buffer) at room temperature using a mica substrate affixed to the bottom of a small Petri dish to ensure complete submersion and minimize evaporation. The surface was coated with 100 μL poly-l-lysine for 20 min, rinsed with ultrapure water, and dried under nitrogen gas stream. Subsequently, 100 μL of sample aliquot was applied, equilibrated for 15 min, gently washed by pipette aspiration, and covered with 1.5 mL buffer.

Transverse stiffness of fibrillin-1 microfibril beads was measured using a DriveAFM instrument (Nanosurf AG) equipped with BL-AC40TS cantilevers (Olympus Corporation; nominal force constant 0.1 N/m, resonance frequency in air 110 ± 35 kHz), calibrated by the thermal method. Force mapping was performed in static mode with 500 pN trigger point over 500 × 500 nm scan areas with 50 × 50 pixel resolution. Young's modulus values were calculated by fitting the Hertz model (spherical tip geometry, Poisson's ratio 0.5) to the recorded force-distance curves in Nanosurf Studio 12 (Nanosurf AG). Resulting height and Young's modulus maps were exported and further processed in Gwyddion [74].

Structural transition events at the bead level were analyzed using a custom Python-based algorithm. Force-distance data were first smoothed using a rolling average (window size: 15 points) to reduce noise. Indentation events were then detected with the SciPy find_peaks function using empirically optimized parameters (prominence: 3 × 10^−12^ N, minimum peak height: 0.5 × 10^−10^ N) to capture distinct deflections while minimizing noise-related artifacts. For each event, the peak indentation force was recorded together with the associated deformation amplitude, defined as the additional indentation required to restore the same force level following the force drop and calculated as the distance (Δx) between the deformation force peak and the corresponding equivalent-force point.

### Statistical analysis

Statistics were performed using GraphPad Prism 8 (GraphPad Software). Outliers were identified using the ROUT method (Q = 1%) and excluded. Depending on sample size, normality was assessed by the Shapiro-Wilk or Anderson-Darling test. Data are presented as mean ± standard deviation or median with interquartile range (IQR). Categorical variables were compared using Fisher's exact test. Group comparisons were conducted using the Kruskal-Wallis test followed by Dunn's post hoc analysis. Correlations were assessed using Pearson or Spearman coefficients. Statistical significance was set at *p* < 0.05.

## Founding sources

This work was supported by grants from the Hungarian National Research, Development and Innovation Office (K150826 and 2020‐2.1.1-ED-2024-00292/EUniWell to M.S.Z. Kellermayer, FK145928 to K. Benke and EKÖP-2025-328 to B. Kiss) and the New National Excellence Program of the Ministry for Culture and Innovation from the source of the National Research, Development, and Innovation Fund (ÚNKP-22-3-I-SE-49 and ÚNKP-23-3-II-SE-22 to C.M. Șulea), as well as the grant of Janos Bolyai Research Scholarship of the Hungarian Academy of Sciences (BO/00242/24 to K. Benke). Projects TKP2021-EGA-23 and RRF-2.3.1‐21‐2022-00003 (National Cardiovascular Laboratory) were implemented with the support provided by the Ministry of Innovation and Technology of Hungary from the National Research, Development and Innovation Fund and the European Union, respectively.

## Declaration of generative AI and AI-assisted technologies in the manuscript preparation process

During the preparation of this work the authors used ChatGPT in order to improve text readability. After using this tool, the authors reviewed and edited the content as needed and take full responsibility for the content of the published article.

## CRediT authorship contribution statement

**Cristina M. Șulea:** Conceptualization, Data curation, Formal analysis, Investigation, Methodology, Resources, Software, Visualization, Writing – original draft, Writing – review & editing. **Dominik Sziklai:** Methodology, Software, Visualization, Writing – original draft, Writing – review & editing. **Bálint Kiss:** Methodology, Visualization, Writing – review & editing. **Kálmán Benke:** Resources, Writing – review & editing. **Miklós Pólos:** Resources, Writing – review & editing. **Bence Ágg:** Data curation, Writing – review & editing. **Roland Stengl:** Data curation, Writing – review & editing. **Máté Csonka:** Writing – review & editing. **Zoltán Szabolcs:** Project administration, Resources, Supervision, Writing – review & editing. **Miklós S.Z. Kellermayer:** Conceptualization, Formal analysis, Funding acquisition, Methodology, Project administration, Resources, Supervision, Validation, Writing – review & editing.

## Declaration of competing interest

The authors declare that they have no known competing financial interests or personal relationships that could have appeared to influence the work reported in this paper.

## Data Availability

The data supporting the findings of this study are included within the article and its Supplementary Material. Additional data underlying the analyses presented in this work are available from the corresponding authors upon reasonable request.

## Glossary

AFM: atomic force microscopy/microscope
BMI: body mass index
C: control
(cb)EGF: (calcium-binding) epidermal growth factor(‐like domain)
DN: dominant negative
EGF: epidermal growth factor(‐like domain)
*FBN1*: fibrillin-1 gene
HI: haploinsufficiency
IQR: interquartile range
MFS: Marfan syndrome
TB: transforming growth factor-beta-binding protein-like (domain)
TGF-β: transforming growth factor-beta

## Appendix A Supplementary data

Supplementary material Table S1. General characteristics and revised Ghent diagnostic criteria of patients with MFS. Table S2. General characteristics of subjects included in the control group.Table S3. Measured fibrillin-1 microfibril bead parameters for each sample. Table S4. Interbead height and periodicity values, and results of interbead height-periodicity correlation tests for all samples. Table S5. Transverse stiffness of aortic fibrillin-1 microfibril beads measured by AFM force mapping, including deformation peak forces and amplitude values for each sample. Figure S1. Scatter plots of measured Young’s modulus values for the included aortic samples.
