# Supplementary material for: Genotype-associated structural and nanomechanical alterations of aortic fibrillin-1 microfibrils in Marfan syndrome
Source: Matrix Biol Plus. 2026 Aug 31;32:100205. doi: 10.1016/j.mbplus.2026.100205 (PMC13563585; doi:10.1016/j.mbplus.2026.100205)
Supplement: Supplementary file 1 [file mmc1.pdf]

Decoding the architecture and mechanics of aortic fibrillin-1 microfibrils in Marfan syndrome

Cristina M. Șulea [a,b,c], Dominik Sziklai [a], Bálint Kiss [a,d], Kálmán Benke [b,c], Miklós Pólos [b,c], Bence Ágg [b,c,e], Roland Stengl [b,c], Máté Csonka [b,c], Zoltán Szabolcs [b,c], Miklós S.Z. Kellermayer [a,d]

[a] Department of Biophysics and Radiation Biology, Semmelweis University, 1094 Budapest, Hungary

[b] Heart and Vascular Center, Semmelweis University, 1122 Budapest, Hungary

[c] Hungarian Marfan Foundation, 1122 Budapest, Hungary

[d] HUN-REN-SE Biophysical Virology Group, 1094 Budapest, Hungary

[e] Department of Pharmacology and Pharmacotherapy, Semmelweis University, 1089 Budapest, Hungary

Corresponding authors: Cristina M. Șulea and Miklós S.Z. Kellermayer

Postal address: Department of Biophysics and Radiation Biology, Semmelweis University, 1094 Budapest, Tűzoltó str. 37-47, Hungary

Email addresses: cristinasulea@gmail.com, kellermayer.miklos@semmelweis.hu

Phone numbers: +36 20 666 3356, +36 20 825 9994

Table S1. General characteristics and revised Ghent diagnostic criteria of patients with MFS.
Abbreviations and symbols: ♀, female; ♂, male; +, present; BMI, body mass index; *FBN1*, fibrillin-1 gene; MFS, Marfan syndrome.

Studied characteristics	MFS1	MFS2	MFS3	MFS4	MFS5	MFS6	MFS7	MFS8	MFS9	MFS10
**Age**	37	17	36	44	28	16	29	50	54	30
**Sex**	♀	♂	♀	♀	♂	♂	♂	♂	♂	♂
**BMI (kg/m^2)**	23.4	16.1	27.8	24.0	21.3	26.0	32.9	23.8	26.0	11.9
**Risk factors for atherosclerosis**										
**Hypertension**	+		+			+	+	+		+
**Hyperlipidemia**								+		
**Diabetes mellitus**										
**Smoking**								+		+
**Obesity (BMI>30.0)**							+			
**Preoperative echocardiographic findings**										
**Aortic root diameter (mm)**	20	26	21	25		26	29	25	24	28
**Sinus Valsalva diameter (mm)**	48	49	41	50	52	46	46	73	47	44
**Ascending aortic diameter (mm)**	46	56	33	37	48	39	40		34	51
**MFS-specific diagnostic criteria (revised Ghent nosology)**										
***FBN1* mutation**	+	+	+	+	+	+	+	+	+	+
**Family history**	+		+	+		+	+		+	
**Aortic involvement**	+	+	+	+	+	+	+	+	+	+
Ectopia lentis			+		+				+	+
**Systemic involvement**	+	+	+	+	+	+			+	+

Table S2. General characteristics of subjects included in the control group.

Abbreviations and symbols: ♀, female; ♂, male; +, present; BMI, body mass index; C, control.

Studied characteristics	C1	C2	C3	C4	C5	C6	C7	C8	C9	C10
**Age**	66	56	67	52	52	43	57	55	56	61
**Sex**	♂	♂	♂	♀	♂	♂	♀	♂	♂	♀
**BMI (kg/m^2)**	29.1	27.8	24.6	25.0	26.3	30.1	28.7	30.6	22.6	29.9
**Risk factors for atherosclerosis**										
**Hypertension**	+	+	+	+	+	+	+	+	+	+
**Hyperlipidemia**	+	+			+	+	+	+	+	+
**Diabetes mellitus**	+	+		+				+	+	
**Smoking**	+	+			+		+			+
**Obesity (BMI>30.0)**						+		+		
**Preoperative echocardiographic findings**										
**Aortic root diameter (mm)**		17	28		21					29
**Ascending aortic diameter (mm)**		28		28	27	37	32			37

Table S3. Measured fibrillin-1 microfibril bead parameters for each sample. The analysis included 97 microfibrils with 2,202 beads in the MFS group and 77 microfibrils with 2,131 beads in the control cohort. For consistency, variables are reported as median with IQR, regardless of distribution.
Abbreviations: C, control; MFS, Marfan syndrome.

Sample ID	Maximum height (nm)	Average height (nm)	Length (nm)	Width (nm)	Projected area (nm^2)	Volume (nm^3)	Roundness	Aspect ratio
**MFS1**	7.46 (6.86–8.27)	4.26 (3.83–5.06)	18.1 (16.4–19.8)	18.6 (17.0–20.4)	640 (487–799)	2,773 (2,327–3,227)	0.64 (0.58–0.68)	1.02 (0.91–1.19)
**MFS2**	8.70 (7.74–9.81)	5.20 (4.51–6.37)	16.9 (14.6–18.4)	18.4 (16.6–19.6)	586 (489–705)	3,015 (2,467–3,882)	0.63 (0.58–0.69)	1.10 (0.96–1.27)
**MFS3**	7.22 (6.60–7.70)	4.52 (4.32–4.79)	18.7 (16.2–20.4)	20.2 (18.3–22.3)	574 (471–712)	2,624 (2,173–3,157)	0.63 (0.57–0.69)	1.08 (0.92–1.29)
**MFS4**	9.13 (8.22–10.00)	5.77 (5.00–6.45)	18.9 (17.5–20.1)	19.5 (18.2–20.9)	653 (551–752)	3,571 (3,144–4,009)	0.66 (0.60–0.69)	1.04 (0.93–1.13)
**MFS5**	8.24 (7.63–9.07)	4.36 (4.12–4.68)	18.8 (16.9–20.6)	19.6 (17.9–22.0)	700 (593–818)	3,132 (2,602–3,663)	0.63 (0.57–0.67)	1.04 (0.92–1.25)
**MFS6**	8.20 (7.67–8.72)	4.87 (4.45–5.36)	19.2 (17.8–20.7)	19.7 (18.3–21.7)	725 (637–820)	3,438 (2,967–4,078)	0.59 (0.54–0.64)	1.03 (0.93–1.15)
**MFS7**	7.49 (6.79–8.14)	4.94 (4.39–5.51)	19.4 (18.0–21.3)	20.9 (19.2–22.4)	651 (570–782)	3,343 (2,725–4,023)	0.69 (0.64–0.73)	1.07 (0.95–1.17)
**MFS8**	10.3 (9.68–10.8)	6.26 (5.92–6.83)	19.1 (17.7–20.5)	19.9 (18.4–21.5)	573 (496–669)	3,633 (3,136–4,278)	0.67 (0.62–0.71)	1.04 (0.92–1.16)
**MFS9**	9.06 (8.38–10.4)	5.83 (5.41–6.69)	19.5 (18.3–20.9)	20.3 (18.9–21.7)	601 (522–668)	3,549 (3,022–4,133)	0.67 (0.62–0.71)	1.04 (0.93–1.14)
**MFS10**	6.44 (5.87–7.07)	3.64 (3.43–3.94)	19.3 (18.0–20.1)	19.9 (18.8–20.9)	972 (853–1,144)	3,534 (3,079–4,157)	0.66 (0.61–0.71)	1.04 (0.97–1.10)
**C1**	6.35 (5.89–6.95)	3.76 (3.52–4.05)	23.6 (22.7–24.2)	23.7 (22.9–24.4)	1,090 (943–1,286)	4,102 (3,319–5,096)	0.71 (0.67–0.74)	1.01 (0.97–1.05)
**C2**	7.82 (7.22–8.34)	4.31 (3.93–4.58)	23.0 (21.8–24.6)	23.7 (22.0–25.4)	976 (841–1,107)	4,076 (3,558–4,644)	0.64 (0.59–0.69)	1.03 (0.94–1.11)
**C3**	8.43 (7.80–9.16)	4.83 (4.48–5.10)	24.3 (22.4–26.0)	24.7 (23.5–26.2)	915 (804–1,082)	4,296 (3,576–5,383)	0.67 (0.63–0.71)	1.04 (0.93–1.12)
**C4**	9.24 (8.54–9.69)	5.38 (4.98–5.67)	23.3 (21.8–24.8)	24.2 (22.6–26.1)	898 (779–1,029)	4,780 (4,103–5,552)	0.63 (0.59–0.66)	1.04 (0.95–1.15)
**C5**	6.70 (6.29–7.48)	4.01 (3.72–4.63)	23.9 (22.7–25.2)	24.5 (23.5–25.8)	1,029 (878–1,181)	4,272 (3,549–4,972)	0.64 (0.60–0.68)	1.02 (0.96–1.10)
**C6**	7.40 (6.70–8.81)	4.40 (3.93–5.47)	23.1 (22.3–24.1)	23.4 (21.9–24.6)	922 (805–1,046)	4,188 (3,545–4,943)	0.66 (0.61–0.70)	1.00 (0.94–1.07)
**C7**	6.47 (6.01–7.16)	4.02 (3.83–4.39)	24.1 (23.0–25.2)	25.8 (24.2–27.2)	1,079 (897–1,236)	4,295 (3,653–5,073)	0.66 (0.62–0.70)	1.06 (0.98–1.15)
**C8**	5.51 (5.05–6.30)	3.53 (3.11–4.01)	23.5 (22.0–24.7)	24.4 (23.1–26.1)	1,189 (1,017–1,362)	4,084 (3,604–4,969)	0.67 (0.62–0.71)	1.04 (0.99–1.12)
**C9**	3.95 (3.65–4.19)	2.50 (2.37–2.66)	24.9 (23.7–26.3)	27.1 (25.4–28.6)	1,802 (1,630–1,996)	4,538 (4,043–5,026)	0.65 (0.61–0.70)	1.08 (1.00–1.15)
**C10**	6.95 (6.57–7.53)	3.68 (3.36–3.93)	23.3 (22.3–24.2)	23.7 (23.0–24.8)	1,153 (1,034–1,307)	4,218 (3,554–4,895)	0.63 (0.58–0.67)	1.01 (0.97–1.09)

Table S4. Interbead height and periodicity values, and results of interbead height-periodicity correlation tests for all samples. For consistency, variables are reported as median with IQR, regardless of distribution.
Abbreviations: C, control; MFS, Marfan syndrome.

			Correlation analysis	
**Sample ID**	**Interbead height (nm)**	**Periodicity (nm)**	**Spearman's r value**	**P value**
**MFS1**	1.10 (0.87–1.38)	52.4 (48.7–55.8)	-0.381	<0.001
**MFS2**	1.06 (0.81–1.44)	49.9 (45.4–52.5)	-0.570	<0.001
**MFS3**	1.09 (0.93–1.29)	55.0 (50.0–61.7)	-0.426	<0.001
**MFS4**	1.28 (0.99–1.79)	52.7 (46.3–58.2)	-0.486	<0.001
**MFS5**	1.33 (0.99–1.91)	52.3 (46.6–57.2)	-0.463	<0.001
**MFS6**	1.66 (1.10–2.48)	48.1 (38.9–56.4)	-0.686	<0.001
**MFS7**	1.43 (1.05–2.00)	49.3 (43.5–55.4)	-0.660	<0.001
**MFS8**	1.39 (1.08–1.77)	51.2 (46.0–56.8)	-0.469	<0.001
**MFS9**	1.17 (0.92–1.49)	52.7 (48.1–57.5)	-0.163	0.018
**MFS10**	1.74 (1.34–2.19)	50.8 (45.2–55.4)	-0.602	<0.001
**C1**	1.44 (1.16–1.86)	54.3 (49.6–57.5)	-0.359	<0.001
**C2**	1.21 (0.95–1.53)	52.7 (48.3–58.3)	-0.498	<0.001
**C3**	1.25 (0.91–1.85)	52.8 (48.4–57.6)	-0.201	0.003
**C4**	1.45 (1.18–1.76)	52.8 (47.4–58.5)	-0.277	<0.001
**C5**	1.37 (1.10–1.74)	50.7 (45.6–55.9)	-0.284	<0.001
**C6**	1.39 (0.99–1.89)	53.0 (47.1–57.3)	-0.375	<0.001
**C7**	1.26 (0.92–1.80)	54.7 (49.4–59.5)	-0.475	<0.001
**C8**	1.67 (1.29–2.14)	53.7 (47.4–58.6)	-0.488	<0.001
**C9**	1.60 (1.30–1.95)	52.8 (47.5–57.0)	-0.588	<0.001
**C10**	1.63 (1.22–2.00)	51.1 (45.9–55.8)	-0.588	<0.001

Table S5. Transverse stiffness of aortic fibrillin-1 microfibril beads measured by AFM force mapping, including deformation peak forces and amplitude values for each sample.
Abbreviations: C, control; MFS, Marfan syndrome.

	Bead stiffness assessment		Bead indentation analysis		
**Sample ID**	**Analyzed curves (n)**	**Young's modulus (MPa)**	**Number of peaks (n)**	**Deformation peak force (pN)**	**Deformation amplitude (nm)**
**MFS1**	331	2.28 (1.52–3.32)	23	264 (197–299)	1.83 (1.67–2.23)
**MFS2**	381	1.96 (1.37–2.73)	32	154 (91–244)	1.98 (1.62–2.50)
**MFS3**	305	2.18 (1.60–3.10)	57	170 (128–286)	2.12 (1.74–2.76)
**MFS4**	432	2.51 (1.92–3.17)	53	171 (121–227)	1.96 (1.67–2.37)
**MFS5**	361	2.43 (1.71–3.39)	46	186 (102–303)	2.15 (1.60–2.93)
**MFS6**	318	2.92 (2.17–3.73)	65	186 (102–273)	1.95 (1.53–2.74)
**MFS7**	342	2.59 (2.05–3.13)	57	167 (103–249)	2.27 (1.83–2.94)
**MFS8**	226	2.58 (1.81–3.62)	78	136 (89–213)	2.14 (1.77–2.85)
**MFS9**	305	3.16 (2.44–4.05)	21	240 (181–416)	1.90 (1.57–2.16)
**MFS10**	257	3.21 (2.35–4.57)	59	218 (135–300)	2.03 (1.75–2.46)
**C1**	322	3.40 (2.61–4.37)	20	271 (206–347)	1.93 (1.47–2.69)
**C2**	382	2.53 (1.81–3.29)	33	207 (135–313)	2.06 (1.65–2.81)
**C3**	431	2.63 (1.94–3.47)	46	267 (212–369)	2.25 (1.75–2.85)
**C4**	348	2.70 (2.01–3.41)	98	235 (154–332)	2.26 (1.75–3.15)
**C5**	203	2.75 (1.97–3.54)	62	191 (127–336)	2.58 (2.11–4.31)
**C6**	315	2.67 (1.77–3.47)	67	200 (152–307)	2.11 (1.58–2.82)
**C7**	325	2.63 (1.87–3.58)	34	215 (139–334)	2.01 (1.65–2.63)
**C8**	304	2.75 (1.90–3.70)	62	301 (225–365)	2.05 (1.61–3.02)
**C9**	217	3.12 (2.11–4.61)	87	245 (160–339)	2.34 (1.73–3.10)
**C10**	373	3.00 (2.10–3.80)	70	289 (186–376)	1.96 (1.60–2.73)

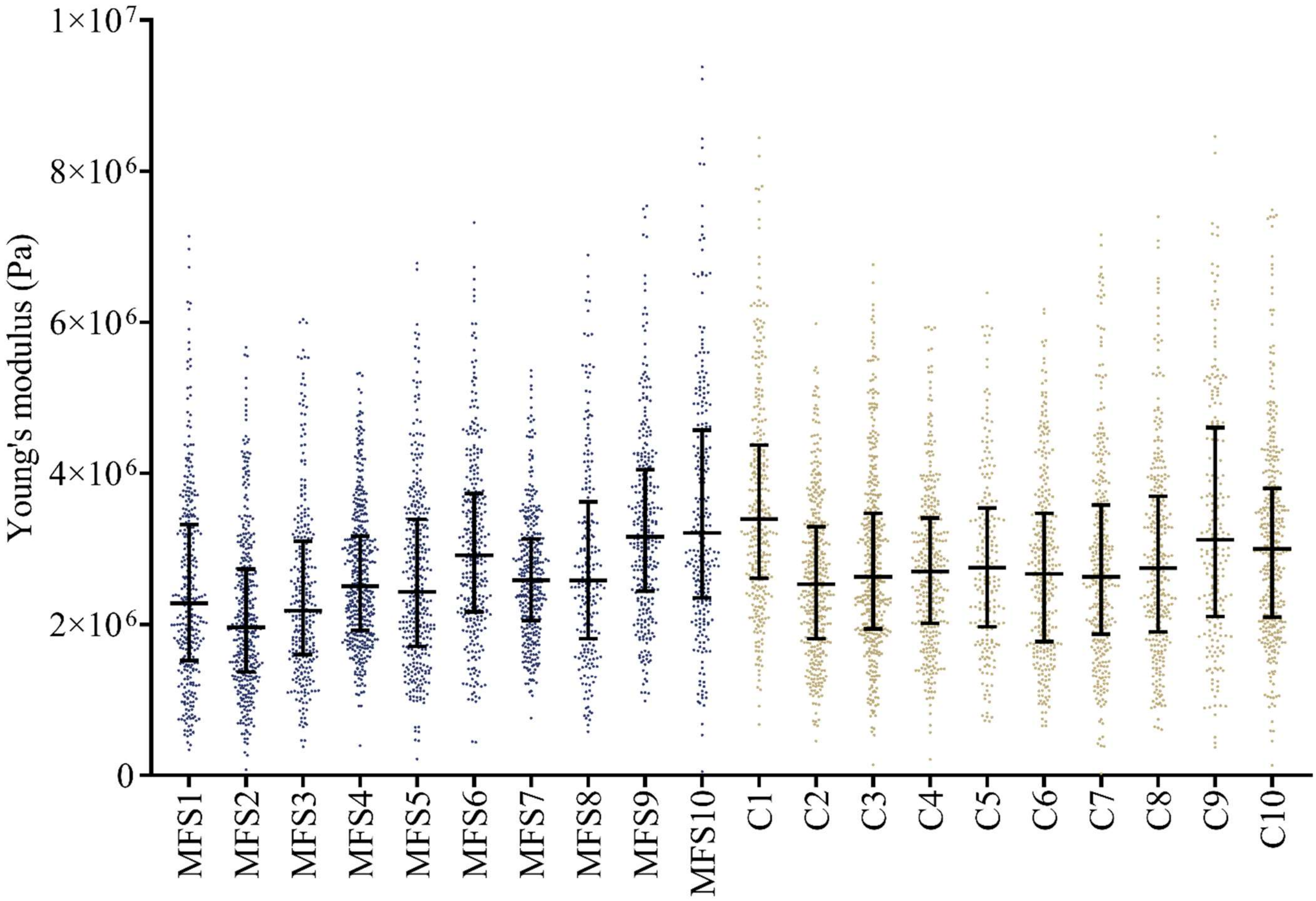

Figure S1. Scatter plots of measured Young's modulus values for the included aortic samples.

Abbreviations: C, control; MFS, Marfan syndrome.

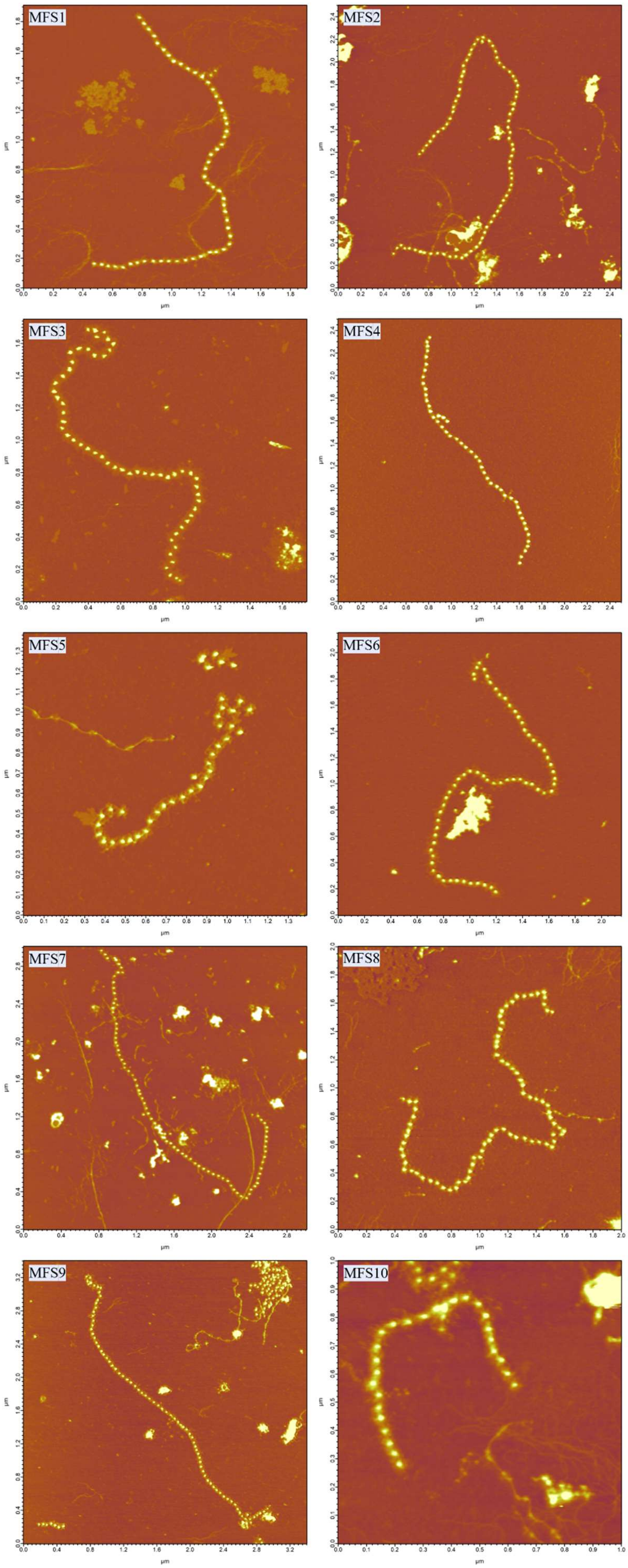

Figure S2. Representative AFM topographical images of fibrillin-1 microfibrils from individual MFS samples.

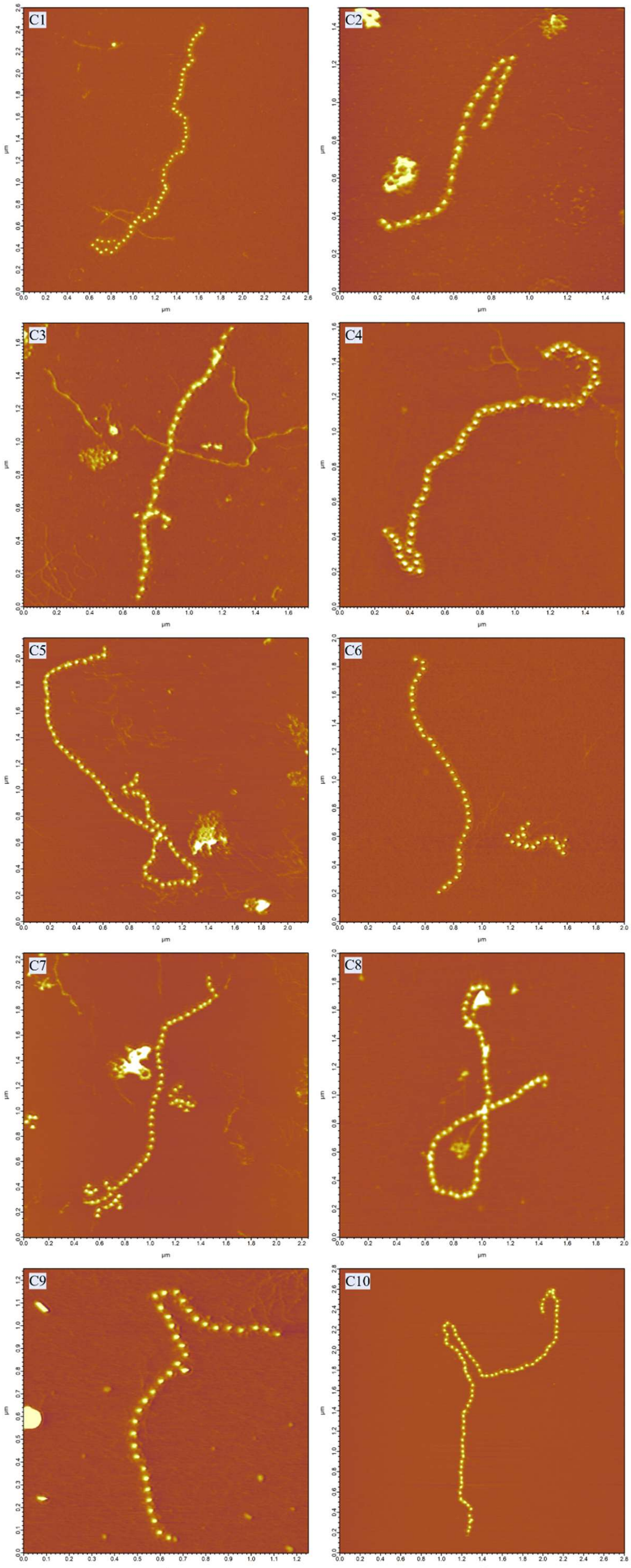

Figure S3. Representative AFM topographical images of fibrillin-1 microfibrils from individual control samples.
